# Ron's Place: the theatre of (personal) power

**DOI:** 10.1017/S2045796022000105

**Published:** 2022-05-04

**Authors:** Lisa Slominski

**Affiliations:** Slominski Projects, London, UK

‘A Greek theatre presupposes tragedy and comedy, and by extension of the presence of the city's people and their allegiance to their heroes and gods. In theatrical space, music, choruses, masks, tiering - all such elements converge with language and actors. A spatial action overcomes conflicts, at least momentarily, even though it does not resolve them; it opens a way from everyday concerns to collective joy.’

– Henri Lefebvre

The extraordinary art environment created privately in a rented flat over decades by Ron Gittins, better known as Ron's Place, is like a personal stage set. Powerful, symphonic, and at times jarring, dimensional scenography occupies every surface, corner and ceiling. Gittins' domestic space became his theatrical platform, where traditional painting confined to canvas or sculptures existing as solitary objects would not suffice his creative vision. Ron's Place is abundant with renderings of ancient pharaohs, emperors and imagery of powerful past civilisations. Sculptural replicas of helmets, chest plates, rifles, swords and dismembered body parts echo military battles throughout history. The concept of power itself was important to Gittins. His niece Jan commented that her uncle ‘was always obsessed with power’ (Hogarth, 2021). While this certainly appears evident in his references to war, military and reign, his intrigue for power could also be considered through a personal lens, in his assertions of agency and control over the transformation of his domestic space. Gittins, in considering his flat a theatre, was all-powerful as its director, producer and lead actor.

**Fig. 1.**
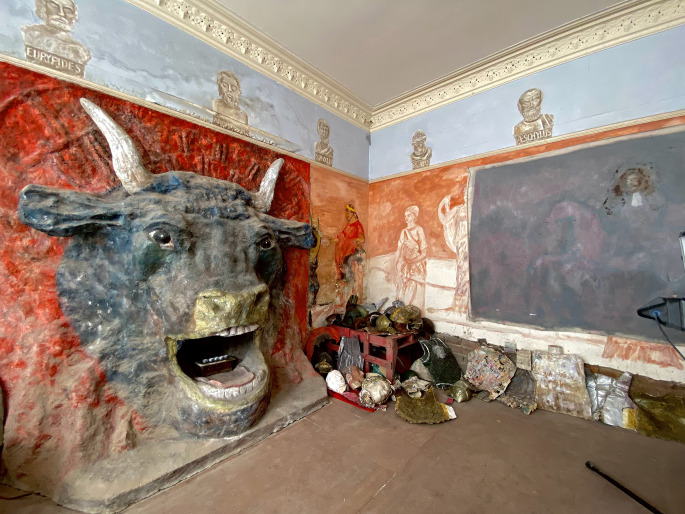
Ron's Place, view of the minotaur in Greek-inspired room, 2021. Photo credit: Cathy Ward for brutjournal

Ron Gittins was born in Birkenhead, a Northern English town across the River Mersey from Liverpool in 1939. He was creative from a young age in theatre, music and visual arts (even briefly attending Laird School of Art). His interest in historical power also emerged during his youth. Gittins' sister noted him sculpting small soldiers from plasticine, detailing their uniforms from distinct global regiments with great detail (Davis, 2020). In 1975, while living with his parents, he transformed his bedroom into a Roman Villa. This precursor to his magnum opus received local press attention. Family and friends noted that mental health issues were present for Gittins throughout his life. His character could be both flamboyant and eccentric, but also difficult and erratic. Gittins had respect for particular powers and authorities including the law and the queen; however, he struggled under the direction of others and to operate within set boundaries. Therefore, while he worked several jobs throughout adulthood, employment was limited and he lived in relative poverty. He was known locally for bombastic performative activities – strolling his neighbourhood while adorned in costume and reciting Shakespeare loudly on the streets – however, the creative transformation of his Wirral flat (rented from 1986) was kept significantly private until his unexpected death in September 2019.

**Fig. 2.**
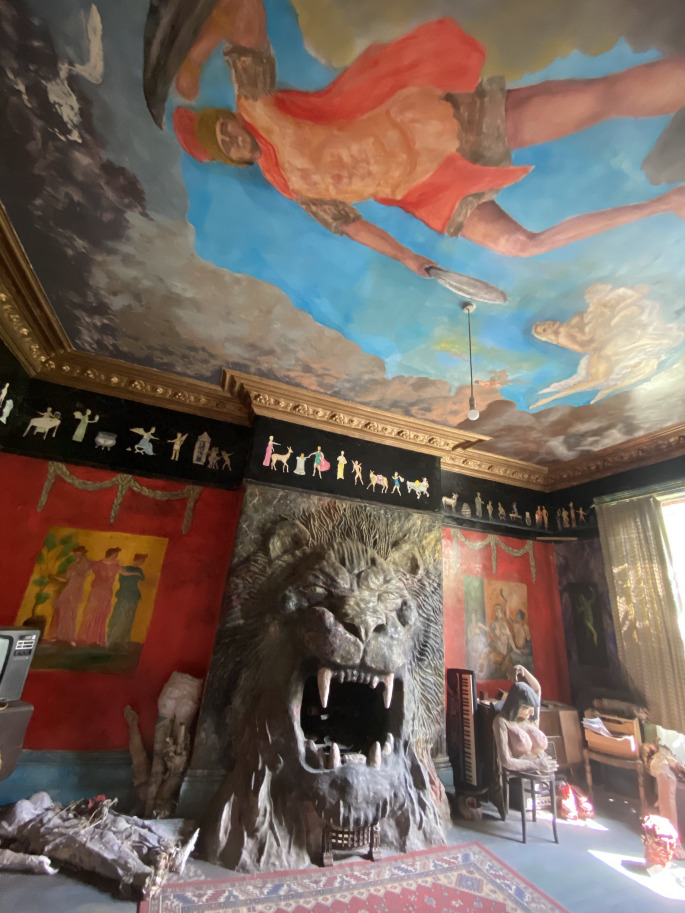
Ron's Place, view Roman-inspired room, 2021. Photo credit: Cathy Ward for brutjournal

After Gittins' death, the majority of his creative modifications have been preserved, while the condition of Ron's Place (the flat itself) still requires repairs. When stepping over the threshold of 8 Silverdale Rd, the vibrant murals on cracked and chipped walls immediately contribute to the comedy, tragedy and drama of theatre.^1^ Though Gittins no longer occupies that space physically, the spatial activity still has an aura of performance and creativity. This consideration of aura or theatrical experience is apropos for visiting Ron's Place.^2^ Each room assumes the role of a carefully constructed backdrop but also feels like entering a play mid-act, unaware if outsiders (us) are extras or trespassers. His flat is a cacophony of the past and present; fact and fiction, the personal and universal; morphing ancient/cultural history with his personal experience. This exploration of Ron's Place is in the spirit of thoughtful interpretation rather than conclusiveness.

**Fig. 3.**
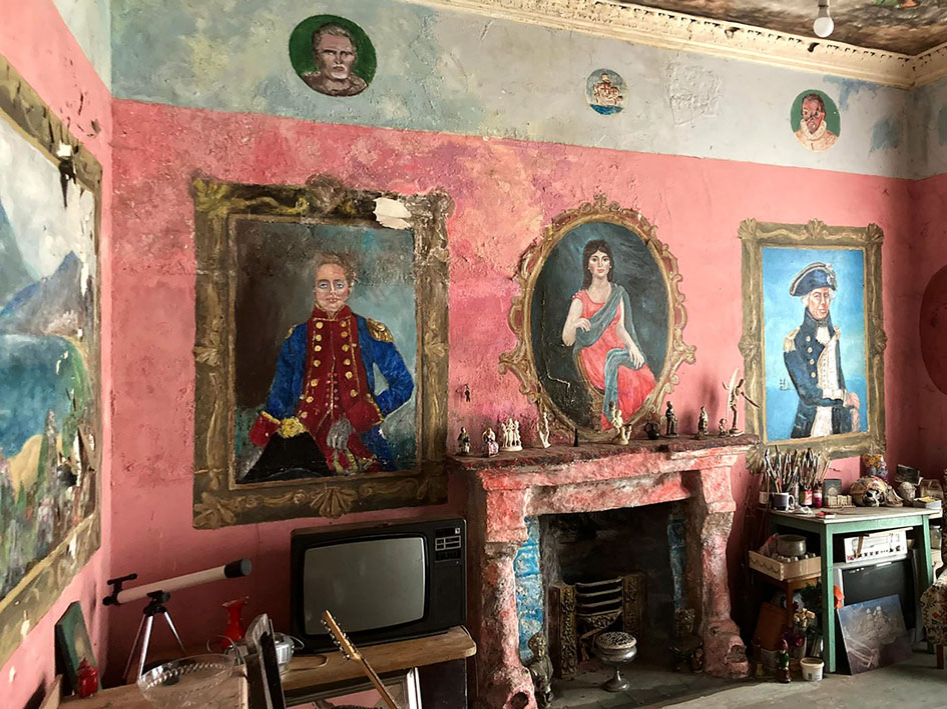
Ron's Place, view of three portraits in Georgian-inspired room, 2021. Photo credit: Cody Ledvina

Hieroglyphics are one of the first visual references when entering Ron's Place, with the entrance/main hallway primarily Egyptian in concept. Here ancient symbols, depictions of the god Anubis, King Tut, Cleopatra and the Sphinx, have been meticulously rendered. Noticeable immediately in the hallway but pertinent to the entire environment is that Gittins' creative actions teetered between replicating architectural adornments and gestures that were pure artistic representations. For example, the hallway features a painted monochromic frieze of hieroglyphics and ancient relics akin to the scenery in the classic film ‘The Ten Commandments’ juxtapositioned with a vibrant full-colour Egyptian Queen with erotic undertones. Other styles are evocative of wall drawings found in an ancient tomb. Some could mistakenly equate the mix-match styles as amateurism, while a more apt consideration could be that Gittins' diverse aesthetic captures the fluidity of historical presentation.

**Fig. 4.**
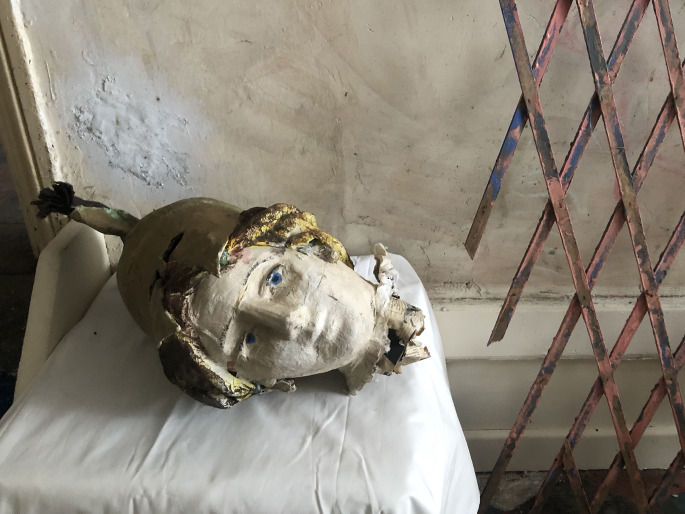
Ron's Place, detail of papier-mache head, 2021. Photo credit: Credit: Lisa Slominski

Moving through the flat, a divergent array of papier-mache helmets and accessories are noticeable in the left front room, piled in the corner. Some appear 20th-century military, others Greco-Roman, and a few have been left in their infancy, only exposing their layers of newspaper. Though Ron's Place has been subject to organisation and removal by those leading the preservation legacy, the remaining assorted volume speaks to an assumed intensity Gittins had for producing a theatrical aura.^3^ His intrigue into power also seems evident in the subject matter of battle helmets, and an array of scrupulously replicated weapons: Roman swords, civil-war muskets and Western rifles. His astuteness for historical accuracy is apparent in the worn books throughout the flat, including *The Art of War in the Western World* by Archer Jones (1987) and *Greek & Roman Mythology* by Malcolm Couch (1997). These few details lend a glimpse into the inner workings of Gittins' process, but also provide consideration for the performative potential in his practice.^4^

The most predominant visual in the room is an immense 3M high sculpture of a Minotaur, a bull-like character integral to Greek mythology. The concrete relief was erected directly from the wall, overtaking the pre-existing architecture. Shaped with a shovel and trowel, its mouth was moulded around a fireplace. Its dominating presence sets the theatrical aura of ancient Greece accentuated by amputated limbs and costumed chest plates scattered through the room. The backdrop of this ancient Greco-room stylistically mimics fresco with scenes (unfinished) of emperors and philosophers. The decorative frieze-like border honours specific Greek historical figures, including the philosopher Plato and ancient playwrights Euripides, and Sophocles. Homage to these Greek writers, known as tragedians for their compositions based on human suffering, demonstrates Gittins' interest in tragedy. Conversely, he was also an admirer of comedy, with a mass of VHS tapes of 1970s British television comedies (found but removed from his flat) (Ward, 2021).

Across the hallway, a second mammoth sculpture of a roaring lion employs another pre-existing fireplace with ferocious teeth clenched around the hearth cavity. The dramatics of this room are predominantly Roman; perhaps the lion is in reference to *damnatio ad bestias*, the ancient Roman act of punishing the condemned by being attacked by wild beasts. Looking up, the painted ceiling inspires a moment of awe. Seemingly inspired by Michelangelo's Sistine Chapel, a trio of delicate and angelic figures is confronted by a Roman soldier with a shield and sword surrounded by billowing clouds. Consistent with Gittins' penchant for architectural detail, the room's painted cornice resembles villagers completing daily tasks. The villagers' figures are silhouetted by a deep black paint that appears to have been a secondary decision; the black perhaps covers a previous, unsatisfactory background. Whereas the unfinished elements of papier-mache helmets and murals in the adjacent room infer Gittins' innate urge to create until his death, the markings amongst this Roman room suggest his urge for power and control; amending and editing his environment until it was exactly ‘right’. The walls feature a vibrant, almost violent, red with dramatic trompe l'oeil decorative roping. Two isolated murals flank the concrete lion and a third large mural on the main wall indistinguishably reads between a window and a painting. Gittins worked for a tailor previously, skills evident in a well-constructed Napoleonic era Redcoat hanging in an armoire; made distinct with a large ‘£’ stitched on the back. If an element of power for Gittins was to be the actor in his theatrical world, this military uniform would certainly have served as his costume in an authoritative scene. Dimensional objects on view, like the Recoatt, depart from ancient Rome but not of intrigue. An erotic and life-size bust of a dark brunette, possibly another nod to Cleopatra, with ample breasts is posed as if an actress in his play, mid-dance.

The third main room feels English, specifically Georgian. A pale red with cracking caused by apparent dampness covers the walls. A light blue wash for the upper border features a series of exquisite circular portraits as if peering through portholes, one resembling Shakespeare. Three significant portraits are painted directly on one wall; each features an ornate frame demarcating it from the red background. The frames are also signifiers that these portraits are important; each an emblematic character of distinct power. Left, appears to be Arthur Wellesley, 1st Duke of Wellington, and right, British admiral, Lord Nelson, while centre-stage above the fireplace is an oval portrait of women. The portrait could be considered as Lady Emma Hamilton; however, research into Gittins' written archive and insight from his family infer, the woman is from an actual romantic relationship. A mural on the main wall adopts the same decorative detail of a painted frame that captures a more serene scene of boats on the water with a volcano reminiscent of Mount Vesuvius from the Bay of Naples. Gittins' creative decision to create a ‘gallery’ in this room could again be evocative of the transformation of his environment as an exercise of conceptual authority. Another fantastical ceiling mural is featured in this room proliferating the theatrical aura. An almost pointillistic, more expressive, style was used to depict a nautical theme, where our perspective may be from underwater watching a mermaid's hand reach towards a Spartan. The specific personal effects that remain in this room allude to entertainment or perhaps the score of Gittins' opus. This includes several televisions and radios, and a guitar, but also an extensive collection of paintbrushes, allowing us to consider the details of Gittins' process.

Even the bathroom and kitchen were worthy spaces for Gittins' creativity. The theatrical backdrop of his bathroom is an underwater aquatic adventure, suiting for the large bathtub within. Octopi, fish, stingrays and sharks circle in the blue water throughout, while on the ceiling, one can catch a glimpse of the world above water, with fireflies and butterflies against the painted blue sky. In the kitchen, the stove area was transformed into an ancient Roman altar. Sculpted out of concrete with the same precision as the lion and minotaur, it features a detailed version of the Grand Master of the Knights Templar seal, including a detailed rendering of the insignia *SIGILLUM MILITUM XPISTI* (Seal of the Soldiers of Christ). Ephemera, source material, paintings made for interaction, historical references and papier-mache body parts are abundant throughout Ron's Place, and considering that the majority of his personal, gathered and creative holdings were removed from the flat after death, he was undoubtedly prolific.^5^

Experiencing, or considering, Ron's Place through the lens of theatre allows the possibility of appreciating not only the outcome of Gittins' environment but also the traces of his activity and the creative aura produced. He demonstrated a performative proclivity outwardly to his community; however, the theatrical spatial aura referred to by Lefebvre need not the city's people present to be powerful. Gittins' multidimensional practice reverberates within Ron's Place, where like other artists who have transformed their homes, it becomes a ‘cultural container’ presenting a complicated but extraordinary relationship between Gittins and his domestic, transformed space (Umberger, 2007). If the concept of theatre, as a spatial action, provides a moment to overcome conflict and allows us to step away from everyday concerns, Ron's Place could have provided as such to him, and certainly to those of us able to experience it following his death. Oscillating between an idiosyncratic subjectivity and universal offering, the power held in Ron's Place is undeniable.

## Footnotes

1 Gittins tenancy agreement stated that the occupant may: ‘decorate the interior of the property to his own taste and the external porch in classical style without the prior written consent of the Landlord.’ Gittins had also constructed outside the exterior entrance to the flat large totems of sculpted figures representing the Battle of Actium of 31 BCE. Gittins' landlord removed these without consultation shortly after his death.

2 Walter Benjamin's concept of an aura expanded beyond the strictly visual into ‘the idea that the interrelation between subject and environment is a process, something that happens in both space and time and that therefore contains a historical dimension.’

3 Saving Ron's Place is an active initiative lead by Jan Williams (Ron's niece) and Chris Teasdale of The Caravan Gallery to preserve Ron's Place and Gittins' legacy for future generations. I thank Professor Iain Jackson and filmmaker Martin Wallace, both on the advisory board, for introducing me to Ron's Place and as a resource for this text. To learn more about the Ron's Place and how to get involved in its preservation visit: https://ronsplace.co.uk/support-us/.

4 There is debate amongst art critics with the use of the term ‘art practice’; however considering the history, transformation and process of Ron's Place, in Gittins' case I consider it apt.

5 The majority of Gittins' written material is currently being archived to allow future/further study.

## About the author

Lisa Slominski is an American curator, writer and cultural producer based in East London. She advocates for neurodiversity in the contemporary art current discourse, and the historical framework and placement of artists considered ‘self-taught’ or ‘outsiders’. In 2020, she co-founded the inclusive international curatorial platform Art *et al*. that focuses on international commissions and collaboration between arts professionals with and without disability. Previously, she curated the first series of public art commissions on Chicago's mass-transit system with the city's Department of Cultural Affairs, was the curator for the project space Tenderpixel, and served as the Head of Visual Culture for the Museum of Everything. *Nonconformers*, her first art history book on artists considered self-taught, will be published in 2022 by Yale University Press. Lisa is dedicated to providing a curatorial voice of diversity and inclusivity within the contemporary art context.

Carole Tansella, *Section Editor*
